# HierL2GDTA: Hierarchical Interaction Modeling with Local and Global Features for Drug–Target Affinity Prediction

**DOI:** 10.3390/molecules31213856

**Published:** 2026-10-26

**Authors:** Kaili Wang, Yaoxiang Yin, Yanglan Gan, Wentao Dai, Zhijun Fang

**Affiliations:** 1 School of Information and Intelligent Science, Donghua University, Shanghai 201620, China; 2 NHC Key Laboratory of Reproduction Regulation, Shanghai Institute for Biomedical and Pharmaceutical Technologies (SIBPT), Fudan University, Shanghai 200237, China; 3 Institute of Trustworthy Embodied AI, Fudan University, Shanghai 200433, China

**Keywords:** drug–target affinity prediction, drug discovery, sequence-based hierarchical framework, modified transformer encoder, intramolecular and intermolecular dependencies, deep learning, target-based drug screening

## Abstract

Accurate prediction of drug–target binding affinity is essential for drug discovery. However, existing methods face limitations in capturing local and global molecular features and in effectively integrating protein and ligand representations for interaction modeling. Here, we present HierL2GDTA, a sequence-based hierarchical framework for predicting binding affinity. Pretrained protein and molecular representations are processed by multi-scale convolutions to extract local features, followed by a bidirectional gated recurrent unit and dilated convolutions to capture broader protein and ligand context, respectively. A modified Transformer encoder integrates global protein context and ligand features through sequential cross-attention to model intramolecular and intermolecular dependencies. HierL2GDTA outperformed the evaluated baseline methods on both BindingDB and Davis. Further evaluations demonstrated the effectiveness of the model components and favorable predictive performance across selected kinase families. Attention visualization highlighted molecular substructures associated with protein binding, while retrospective screening ranked known inhibitors of disease-associated targets among the leading candidates. These results indicate that HierL2GDTA is an effective deep learning method for binding affinity prediction, with potential application for target-based drug screening.

## 1. Introduction

The interaction between proteins and small molecules underlies a wide range of biological processes and constitutes the molecular basis of many therapeutic interventions. Quantifying the strength of such interactions, generally referred to as drug–target binding affinity, is therefore a fundamental task in drug discovery [1]. Accurate binding affinity prediction can facilitate the identification and prioritization of promising compounds during virtual screening, guide lead optimization, and reduce the experimental burden associated with large-scale biochemical assays [2,3]. Experimental measurements, such as the dissociation constant *K_d_*, inhibition constant *K_i_*, and half-maximal inhibitory concentration *IC50*, provide reliable estimates of binding strength but are costly and time-consuming when applied to large chemical libraries. Molecular docking [4] and physics-based free-energy calculations provide computational alternatives, but their practical applications are often constrained by the trade-off between computational efficiency and predictive accuracy [5,6]. Consequently, developing accurate and efficient computational approaches for drug–target binding affinity prediction remains an important challenge in computer-aided drug discovery.

With the rapid development of artificial intelligence, deep learning (DL) has been extensively applied to drug–target binding affinity prediction. According to the types of input representations, current DL methods for drug–target binding affinity prediction [7,8] can be primarily categorized into sequence-based and structure-based approaches.

Sequence-based approaches typically predict binding affinity from protein sequences and ligand SMILES strings without relying on drug–target complex structures [9,10]. For instance, DeepDTA [11] is an early sequence-based affinity model that employs two separate one-dimensional convolutional neural networks (CNNs) to learn representations from protein sequences and ligand SMILES strings. DeepAffinity [12] combined recurrent and convolutional neural networks within a unified framework to capture sequential patterns from protein and compound representations. AttentionDTA [13] used separate 1D CNNs to encode ligand and protein sequences, together with a two-sided multi-head attention mechanism for ligand–protein interaction modeling. More recently, pretrained protein and molecular language models have been increasingly employed in affinity prediction [14]. MutualDTA [15] employs pretrained molecular and protein models to derive context-aware representations, followed by downstream feature extraction and mutual attention-based interaction modeling. Similarly, GeneralizedDTA [16] incorporates pretraining tasks for both molecules and proteins and further fine-tunes the pretrained representations for affinity prediction.

Structure-based approaches explicitly incorporate molecular topology and three-dimensional structural information as input representations [17,18]. Initially, structure-based approaches commonly represented protein–ligand complexes as three-dimensional grids and applied 3D CNNs to learn spatial interaction patterns. Pafnucy [19], for example, encodes protein–ligand complexes into voxelized representations and extracts interaction features through three-dimensional convolutions. Subsequently, graph neural networks (GNNs) have become increasingly powerful and widely used in drug discovery and drug design. GraphDTA [20] represents ligands as molecular graphs and employs GNNs to learn their topological features, while CNNs are used to extract hidden features from protein sequences. Recently, complex-based methods directly construct graphs from 3D protein–ligand complexes. Specifically, PLANET [21] represents the binding pocket as a 3D graph to capture its spatial and structural information. Meanwhile, multimodal frameworks that integrate molecular information across multiple levels have also been increasingly applied to drug–target affinity prediction. In particular, GIF-PLA [22] combines meta-path-enhanced representations of heterogeneous protein–ligand graphs with sequence information to capture higher-order structural and semantic relationships.

Although existing models have achieved promising results in affinity prediction, they still face certain limitations. Structure-based approaches provide direct access to geometrical and intermolecular interaction information, but their performance depends on the availability and quality of the structures. The computational cost also increases with the complexity and level of detail of structural representations. Recent studies have shown that even geometrically plausible protein–ligand poses may fail to recover key intermolecular interactions [23]. Intermolecular Interaction Network Fidelity (I-INF) has been introduced to quantify the agreement between predicted and reference interchain interactions in macromolecular complexes [24], providing a useful basis for evaluating modeled interfaces and informing the development of structure-based interaction prediction models. Sequence-based approaches are more broadly applicable, as they do not explicitly depend on complex structures. However, existing methods remain limited in modeling multi-scale local features and long-range dependencies among different local regions. In addition, the capacity to effectively integrate protein–ligand interaction information is still insufficient.

**Figure 1:**
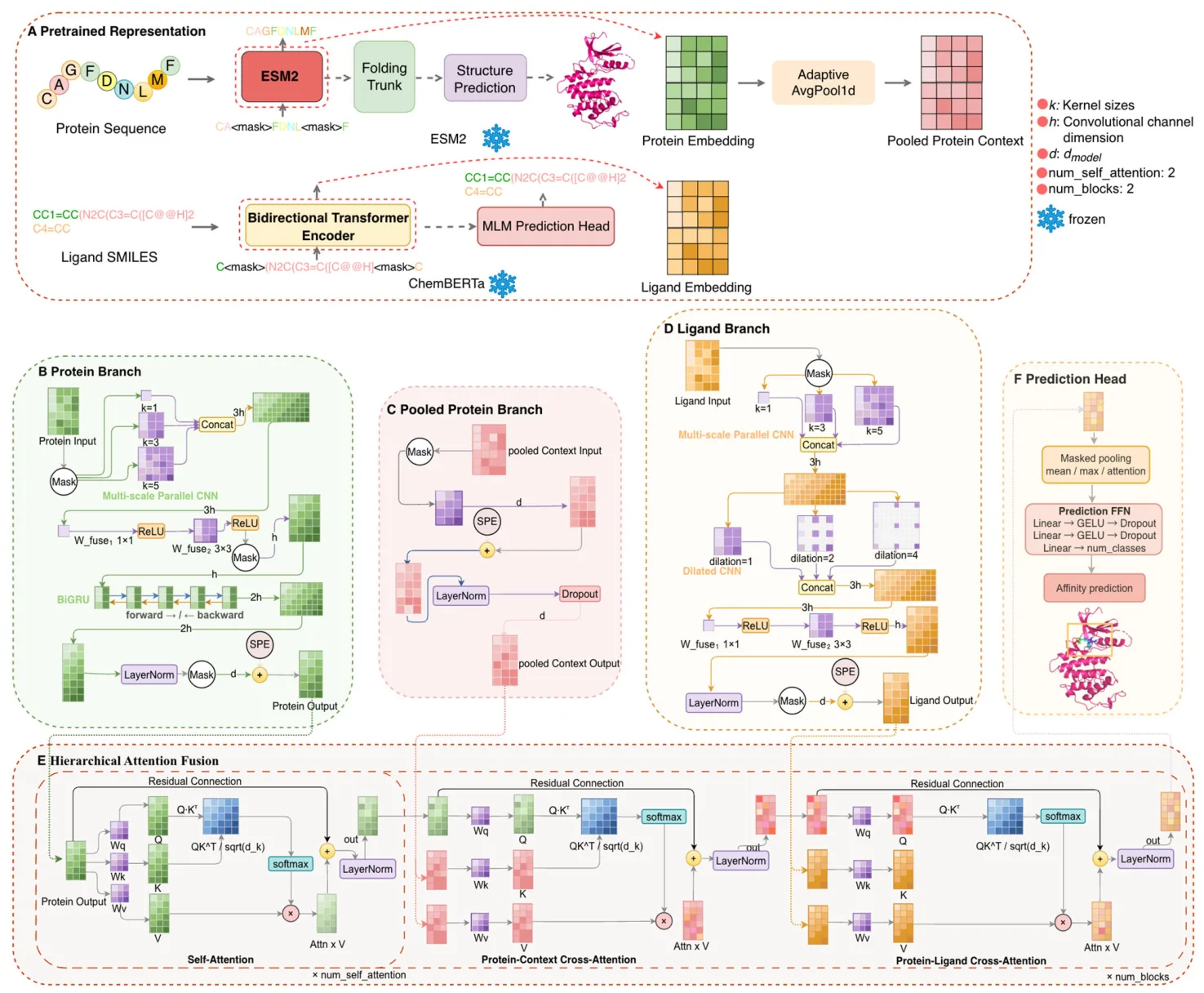
The framework of HierL2GDTA. (**A**) ESM-2 and ChemBERTa encode protein sequences and ligand SMILES, respectively; adaptive average pooling generates a fixed-length protein context. (**B**) Multi-scale convolutions and a BiGRU extract local and long-range protein features, respectively. (**C**) The pooled protein context undergoes projection and positional encoding. (**D**) Multi-scale and dilated convolutions capture local and broader ligand context, respectively. (**E**) Self-attention models intramolecular dependencies, followed by protein-to-context and protein-to-ligand cross-attention for hierarchical fusion. (**F**) Masked attention pooling and an FFN produce the binding affinity prediction.

To address these challenges, we developed HierL2GDTA, a framework for drug–target binding affinity prediction that combines local and global feature extraction with hierarchical modeling of intramolecular and intermolecular interactions. Specifically, pretrained protein and molecular language models are first employed to provide context-aware representations. Multi-scale convolutional modules with different receptive fields are subsequently introduced for both proteins and ligands to capture complementary local patterns at multiple contextual scales. To capture dependencies beyond local patterns, distinct encoding strategies are employed for proteins and ligands. A bidirectional gated recurrent unit (BiGRU) is employed to capture long-range sequential dependencies within proteins, whereas dilated convolutions are introduced for ligands to progressively enlarge the receptive field and characterize broader molecular context without excessively increasing network depth. Finally, a variant of the Transformer encoder is constructed to further refine and integrate the learned protein and ligand representations, allowing the model to capture high-level dependencies associated with intermolecular interactions. The overall architecture of the proposed model is illustrated in Figure 1.

## 2. Results

### 2.1. Parameter Setting

All experiments were implemented in PyTorch 2.13.0 and conducted on a single NVIDIA RTX PRO 6000 GPU with 96 GB of memory. ESM-2 650M and ChemBERTa-77M-MTR were used as the pretrained protein and ligand encoders, with embedding dimensions of 1280 and 384, respectively. Each branch of the multi-scale convolutional modules used 640 output channels. The common representation dimension *d*_model_ was set to 384, and each multi-head attention layer used four heads. The dropout rate was set to 0.15.

The model was optimized using AdamW with a batch size of 150, a learning rate of 1 × 10^−4^, and a weight decay of 1 × 10^−4^. Mean squared error (MSE) was used as the loss function. Gradients were clipped to a maximum norm of 5.0. Training was run for up to 70 epochs on Davis and 100 epochs on BindingDB, with early-stopping patience values of 15 and 20 epochs, respectively. The model checkpoint with the lowest validation MSE was selected for final evaluation. The detailed parameter settings are summarized in Table S1.

### 2.2. Comparison with Competing Methods

**Figure 2:**
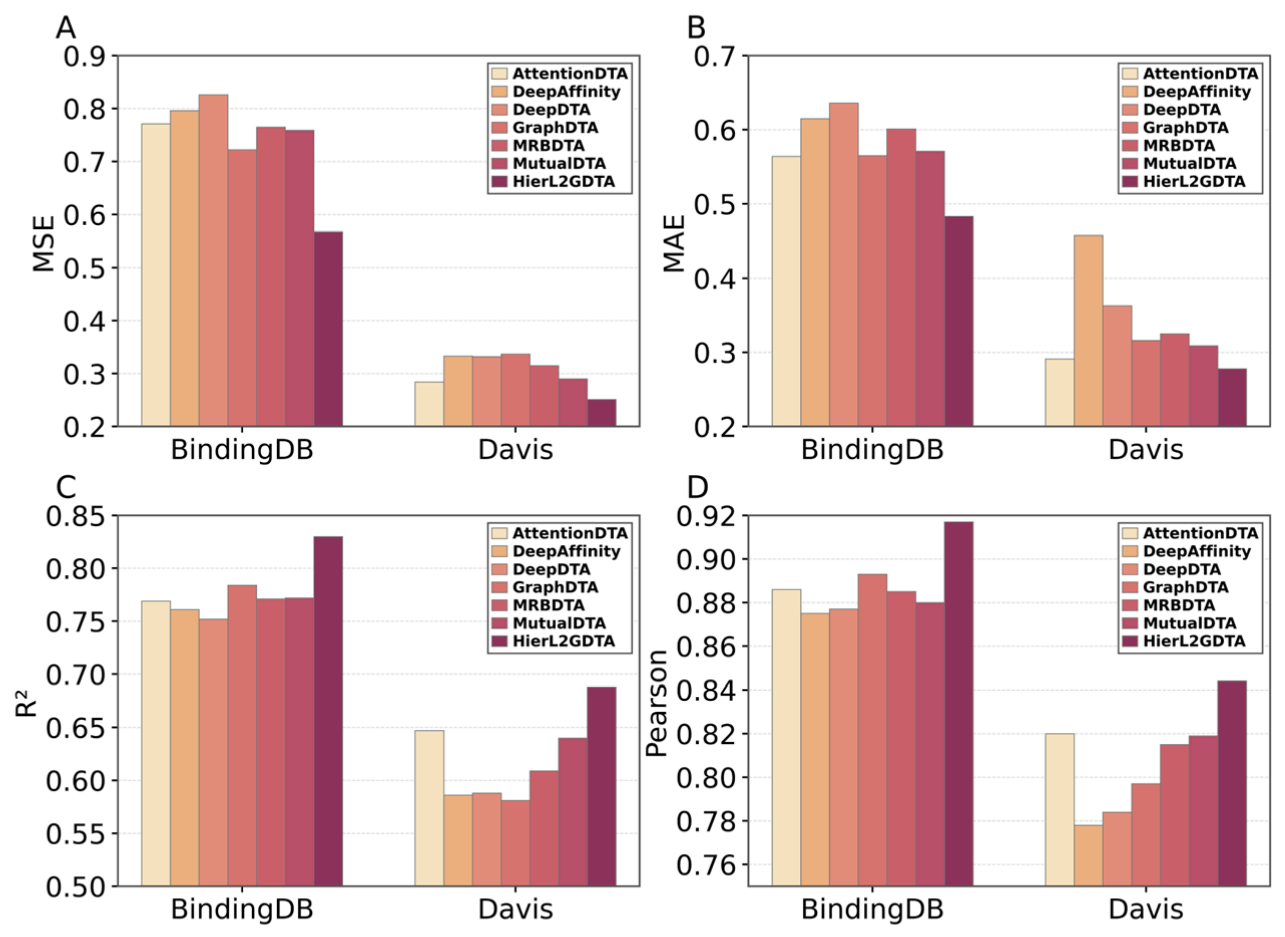
Performance of HierL2GDTA and the competitive methods on BindingDB and Davis test sets for four metrics: (**A**) MSE, (**B**) MAE, (**C**) *R*^2^, and (**D**) Pearson.

HierL2GDTA was evaluated on the training, validation, and test sets of BindingDB and Davis (Table 1), showing comparable performance between the validation and test sets. We further compared HierL2GDTA with six representative drug–target binding affinity prediction methods, including DeepDTA, DeepAffinity, GraphDTA, MRBDTA, AttentionDTA, and MutualDTA. For fair comparisons, all baseline methods were trained, validated, and evaluated using the same data splits as HierL2GDTA, with identical test samples, affinity labels, and evaluation metrics. Table 2 presents the performance comparison on the BindingDB and Davis datasets in terms of MSE, mean absolute error (MAE), root mean squared error (RMSE), coefficient of determination (*R*^2^), Pearson correlation coefficient (Pearson), and Spearman rank correlation coefficient (*ρ*). Lower MSE, MAE, and RMSE values indicate smaller prediction errors, whereas higher *R*^2^, Pearson, and *ρ* values indicate greater explanatory power, stronger linear correlation, and stronger monotonic correlation, respectively. On the BindingDB dataset, HierL2GDTA achieved the best performance across all six metrics, with MSE, MAE, RMSE, R^2^, Pearson, and *ρ* values of 0.568, 0.483, 0.754, 0.830, 0.917, and 0.890, respectively. Compared with the best competing result for each metric, HierL2GDTA reduced MSE, MAE, and RMSE by approximately 21.33%, 14.36%, and 11.29%, respectively, while increasing R^2^, Pearson, and *ρ* by 0.046, 0.024, and 0.013, respectively. HierL2GDTA also achieved the best performance across all metrics on the Davis dataset, with corresponding values of 0.251, 0.278, 0.501, 0.688, 0.844, and 0.677. Compared with the strongest competing method, HierL2GDTA reduced MSE, MAE, and RMSE by approximately 11.62%, 4.47%, and 6.00%, respectively, while increasing R^2^, Pearson, and *ρ* by 0.041, 0.024, and 0.014, respectively. Although the relative reductions in prediction errors were smaller on Davis, improvements were observed across all metrics on both benchmarks (Figure 2).

To further assess the robustness and generalization of HierL2GDTA, we conducted additional experiments using repeated random splits and a protein-based cold split. Specifically, three independent experiments were conducted using different random splits, and the mean and standard deviation of each performance metric across the three runs are reported in Table 3 to assess model stability across different data partitions. The corresponding training and validation loss curves are presented in Figure S1. In addition, a protein-based cold split was adopted to assess the model’s predictive performance on unseen protein targets, as presented in Table 4. For this evaluation, the core framework was retained, while two hyperparameters were adjusted: the maximum protein input length was set to 1024, and masked mean pooling was employed. The random seed was set to 2029, and all other experimental settings remained unchanged. Overall, these experiments further demonstrate the robustness of HierL2GDTA across different random splits and its predictive capability on unseen protein targets.

### 2.3. Ablation Study

**Figure 3:**
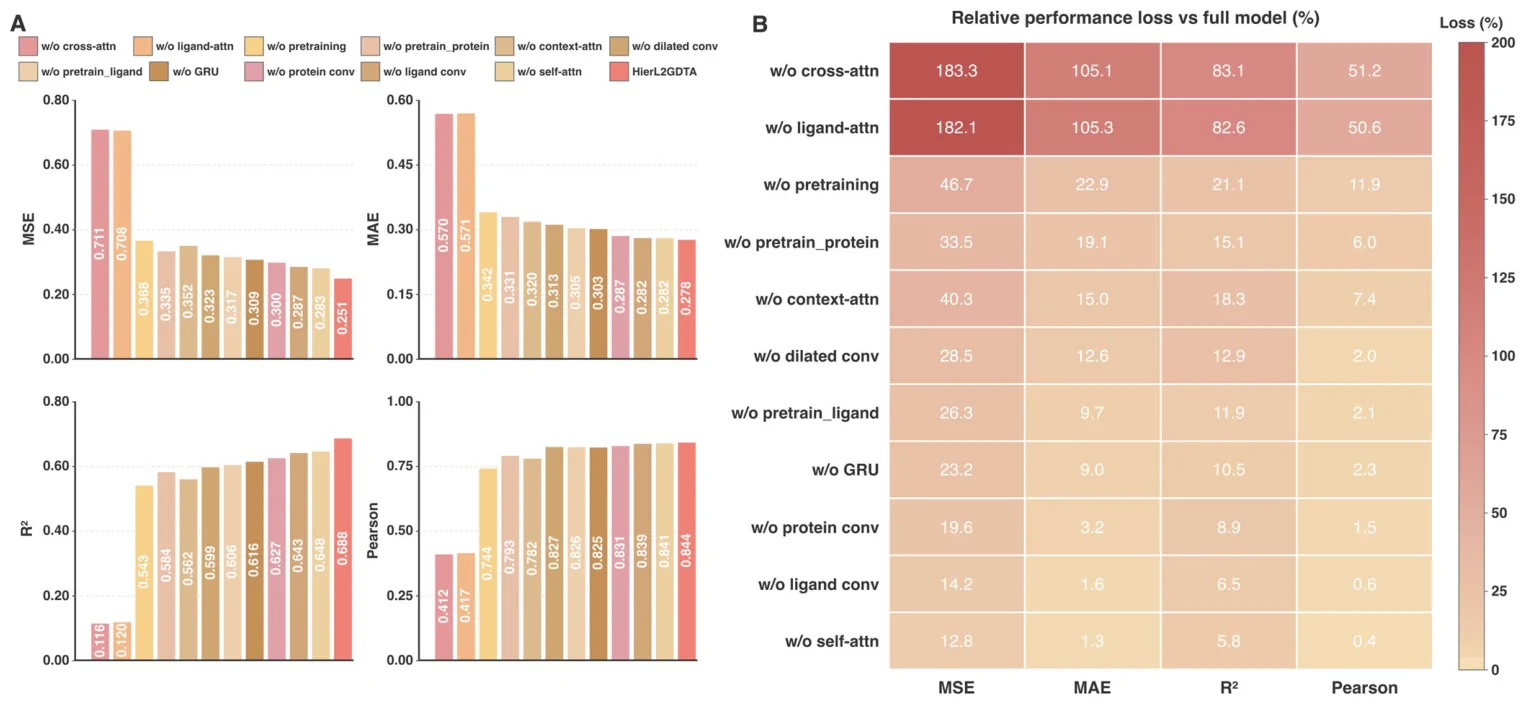
Ablation study of HierL2GDTA on the Davis dataset. (**A**) Performance comparison between the full model and eleven ablated variants. (**B**) Relative performance loss of each ablated variant compared with the full model. Darker shading indicates greater performance degradation.

Eleven ablation experiments were conducted to assess the contributions of key components in HierL2GDTA (Figure 3). Three experiments evaluated the cross-attention modules: w/o cross_attn removed both modules, whereas w/o ligand_attn and w/o context_attn removed protein-to-ligand and protein-to-context cross-attention, respectively. To assess the contributions of pretrained representations, w/o pretraining replaced both protein and ligand representations with one-hot encodings, while w/o pretrain_protein and w/o pretrain_ligand applied the same replacement to protein and ligand representations, respectively. The remaining five experiments separately removed the protein BiGRU (w/o GRU), ligand dilated convolutions (w/o dilated_conv), initial protein self-attention (w/o self_attn), protein multi-scale convolutions (w/o protein_conv), and ligand multi-scale convolutions (w/o ligand_conv). All other experimental settings remained unchanged.

The full model achieved the lowest MSE and MAE and the highest R^2^ and Pearson correlation. Removing both cross-attention modules increased MSE from 0.251 to 0.711 and decreased Pearson from 0.844 to 0.412. Removing protein-to-ligand cross-attention alone resulted in a comparable decline, with an MSE of 0.708 and a Pearson of 0.417. Removing protein-to-context cross-attention also resulted in a notable performance decline, yielding an MSE of 0.352 and a Pearson of 0.782. Replacing both pretrained representations with one-hot encodings increased MSE to 0.368 and reduced Pearson to 0.744. Individually replacing protein or ligand pretrained representations increased MSE to 0.335 and 0.317, respectively, highlighting the importance of both representations. Removing the remaining components caused smaller performance decreases, with MSE values ranging from 0.283 to 0.323. These results indicate that all evaluated components contribute to predictive performance, particularly the two cross-attention modules and pretrained representations.

### 2.4. Parameter Analysis

We conducted a parameter sensitivity analysis on the Davis dataset to evaluate the effects of fusion depth, cross-attention layer allocation, and representation dimension *d*_model_ (Table 5). Two fusion blocks achieved the best overall performance. Fewer blocks reduced predictive performance, whereas additional blocks yielded no consistent improvement. Increasing the number of protein-to-context or protein-to-ligand cross-attention layers individually provided no further benefit.

Performance remained relatively stable across moderate representation dimensions but declined at smaller dimensions. A dimension of 384 provided the best overall balance across metrics, with no consistent improvement at larger dimensions. Accordingly, the final model used two fusion blocks, each containing one protein-to-context and one protein-to-ligand cross-attention layer, with *d*_model_ = 384.

### 2.5. Substructure-Aware Drug Feature Extraction

**Figure 4:**
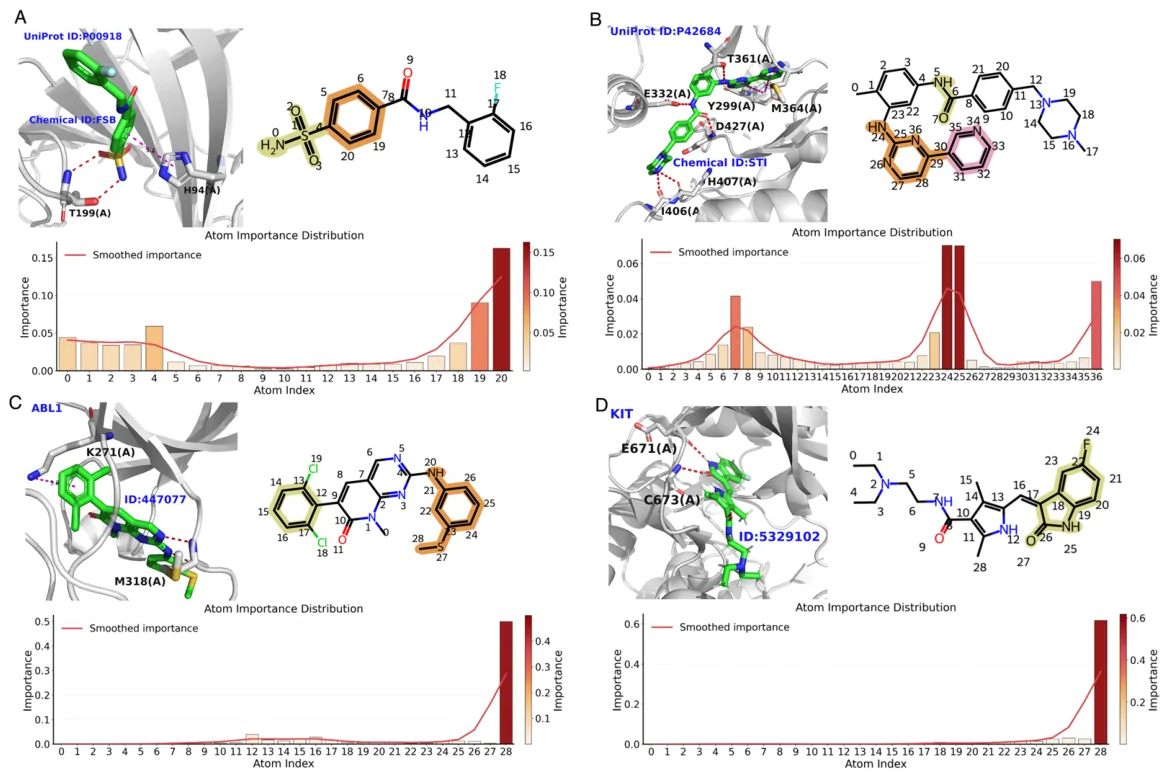
Atom-level attention visualization. Four representative protein–ligand pairs were selected from the BindingDB (**A**,**B**) and Davis (**C**,**D**) test sets: (**A**) P00918–FSB; (**B**) P42684–imatinib (STI); (**C**) ABL1–PD173955 (CID 447077); and (**D**) KIT–sunitinib (CID 5329102). Each panel shows the binding site, indexed ligand structure with highlighted substructures, and atom importance distribution. Proteins are shown in gray and ligands as green sticks; dashed lines indicate selected interactions with labeled residues. Bar heights and colors indicate attention-derived atom importance, with darker colors representing higher scores. Red curves show smoothed importance profiles.

The interpretability of the model was further assessed through atom-level attention visualization (Figure 4). Four protein–ligand pairs were selected from the Davis and BindingDB test sets. For each pair, the 15 highest-ranked ligand atoms were examined in relation to the corresponding binding-site interactions (Table S2). Ligand atom importance scores were derived from the attention weights of the protein-to-ligand cross-attention module. The importance maps highlighted several functional groups and aromatic regions involved in protein binding, providing a structural perspective on the model predictions.

In the carbonic anhydrase II–FSB complex (Figure 4A), high-scoring atoms were located in the sulfonamide group (atoms 0–3) and the attached benzene ring (atoms 4–7, 19, and 20). The sulfonamide group participates in two hydrogen bonds with Thr199, whereas the benzene ring forms a π–π contact with His94. The correspondence therefore extended to both polar and aromatic interactions. A similar pattern was observed for ABL2–imatinib (Figure 4B), where several highly ranked atoms belonged to the substituted 2-aminopyrimidine moiety (atoms 24–29 and 36) and the amide linker (atoms 5–7). These regions participate in hydrogen bonding with Thr361, Glu332, and Asp427, connecting the highlighted substructures to specific contacts within the binding pocket.

Aromatic substructures were also emphasized in the ABL1–PD173955 complex (Figure 4C). All six carbon atoms of the benzene ring (atoms 12–17) were highly ranked, consistent with the cation–π contact identified between this ring and Lys271. Several high-scoring atoms were also located within the 3-(methylthio)anilino group (atoms 20–28), while two hydrogen bonds with Met318 involved the anilino–pyrimidine junction. In the KIT–sunitinib complex (Figure 4D), the highlighted 5-fluorooxindole moiety (atoms 17–27) was fully represented among the top 15 atoms. Its carbonyl oxygen O27 and lactam NH group at N25 participate in hydrogen bonds with Cys673 and Glu671, respectively, linking the importance profile to the hinge-binding region of the ligand.

Although not every atom with a high importance score could be directly associated with an annotated protein contact, as illustrated by C28 in PD173955 and sunitinib, several highlighted substructures were involved in protein–ligand interactions. The identified functional groups and aromatic moieties participate in hydrogen bonding and aromatic contacts, emphasizing the relevance of these substructures to molecular recognition. These observations support the model’s ability to identify binding-relevant substructures, providing a chemically meaningful basis for interpreting its affinity predictions.

### 2.6. Prediction Performance Across Protein Families

**Figure 5:**
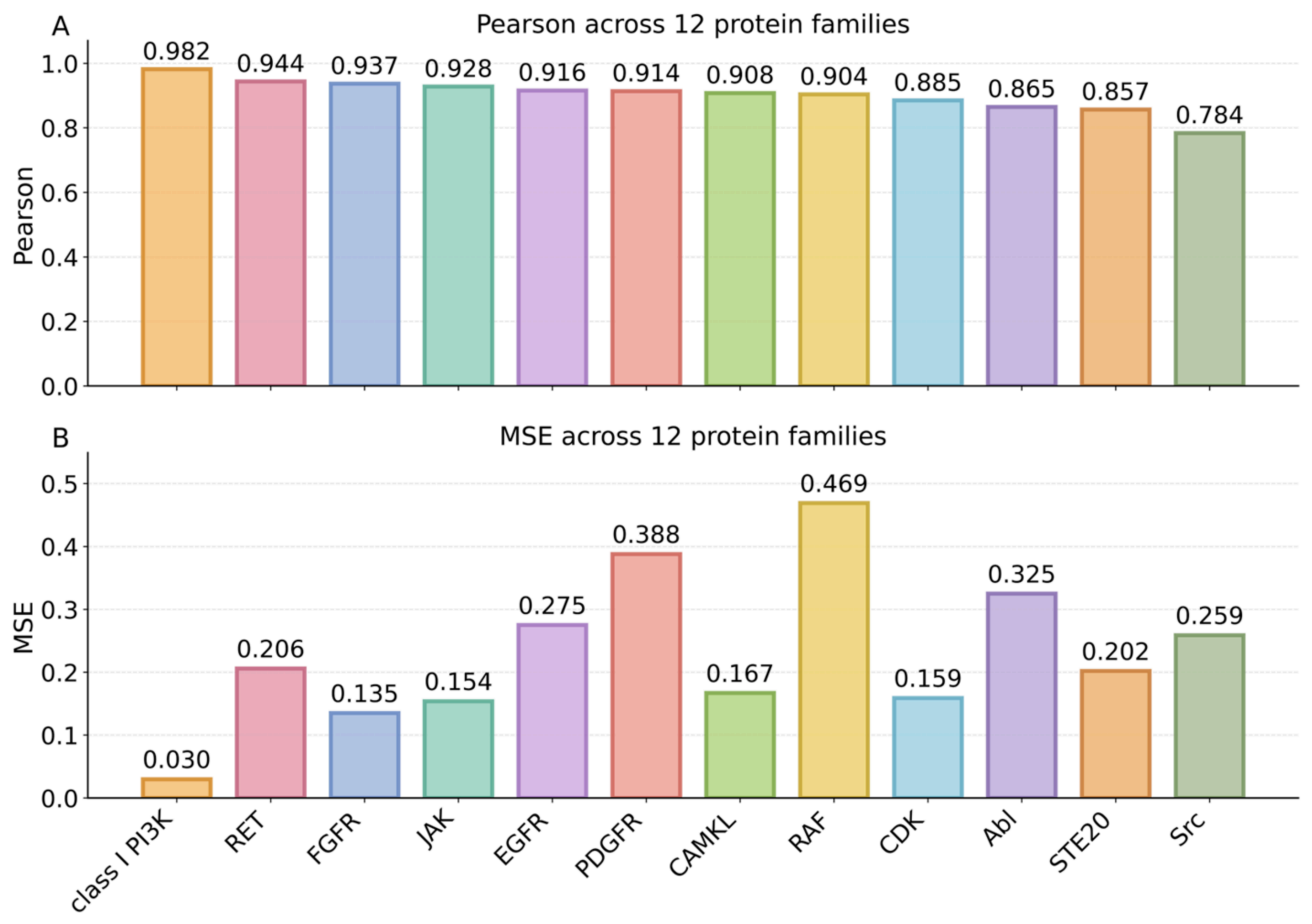
Binding affinity prediction performance of HierL2GDTA across 12 protein families: (**A**) Pearson’s correlation and (**B**) MSE.

Binding affinity prediction was evaluated across kinase families in the Davis test set. Twelve families with at least 20 protein–ligand pairs were randomly selected for analysis (Figure 5). The selected families included EGFR, PDGFR, FGFR, JAK, class I PI3K, RET, CAMKL, CDK, STE20, Abl, RAF, and Src. Strong correlations were observed between predicted and experimental binding affinities, with Pearson correlation exceeding 0.85 for 11 of the 12 families (Table S3). Pearson correlations above 0.90 were obtained for class I PI3K, RET, FGFR, JAK, EGFR, PDGFR, and CAMKL. Among these families, EGFR, PDGFR, and CAMKL comprised 114, 131, and 116 test pairs, respectively, spanning 15, 18, and 19 kinase entries. Correlations of 0.885, 0.865, and 0.857 were obtained for CDK, Abl, and STE20, respectively. STE20 had the largest test subset among the selected families, comprising 188 drug–target pairs across 27 kinase entries, with an MSE of 0.202. For RAF, a high Pearson correlation of 0.904 was accompanied by a comparatively high MSE of 0.469. The affinities of two high-affinity pairs were underestimated, and these predictions accounted for 75.7% of the total squared error, indicating that the elevated MSE was largely attributable to these individual predictions. Src exhibited the lowest Pearson correlation (0.784) among the selected families, with experimental *pK_d_* values spanning a relatively narrow range of 5.000–7.824. These observations suggest that differences in experimental affinity distributions may affect the observed correlations across protein families, highlighting the need to interpret linear correlation coefficients in the context of the underlying data variance. Overall, the model showed favorable predictive performance across the selected kinase families, suggesting that it is useful for estimating drug–target binding affinities within these families.

### 2.7. Compound Prioritization for Disease-Associated Targets

**Figure 6:**
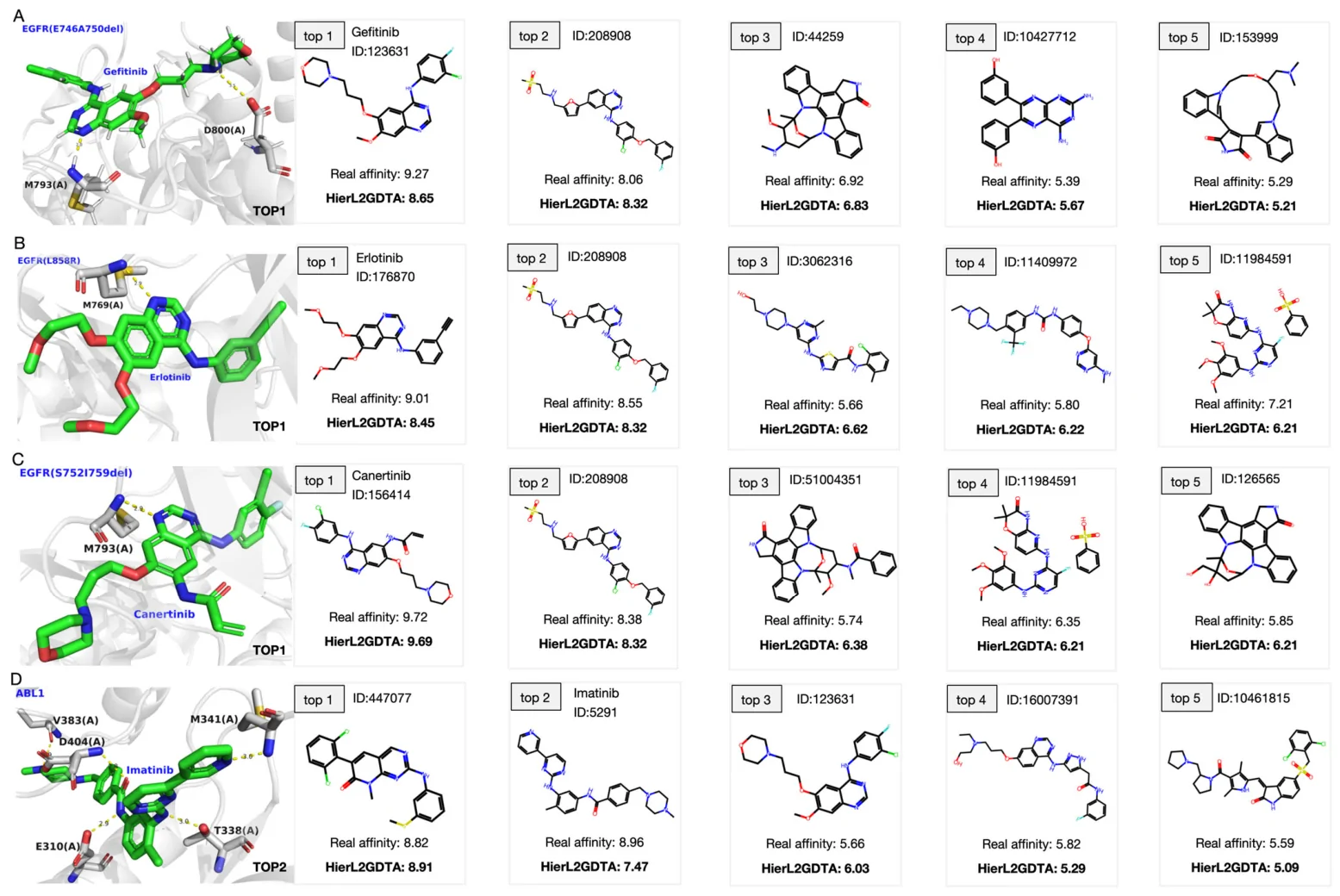
The five highest-ranked compounds predicted by HierL2GDTA within the Davis test involving (**A**) EGFR(E746A750del), (**B**) EGFR proteins harboring L858R, (**C**) EGFR(S752I759del), and (**D**) ABL1. Molecular structures, identifiers, and experimental and predicted (*pK_d_*) values are shown. Binding-site views depict the first-ranked compounds gefitinib (**A**), erlotinib (**B**), and canertinib (**C**), and the second-ranked compound imatinib (**D**). Proteins are shown in gray and ligands as green sticks; dashed lines indicate selected interactions with labeled residues.

The utility of HierL2GDTA for disease-oriented compound prioritization was assessed in a screening analysis (Figure 6). All protein–ligand pairs included in this analysis were obtained from the Davis test set. Candidate compounds were ranked according to their predicted binding affinities within the corresponding target subsets (Table S4). EGFR and its activating variants are therapeutic targets in non-small-cell lung cancer (NSCLC) [25]. Among seven pairs involving EGFR(E746A750del), gefitinib was ranked first, with a predicted *pK_d_* of 8.65 compared with an experimental value of 9.27 (Figure 6A). Similarly, erlotinib was ranked first among 22 pairs involving EGFR proteins harboring the L858R mutation (Figure 6B). Its predicted and experimental *pK_d_* values were 8.45 and 9.01, respectively. Canertinib, an investigational pan-ErbB inhibitor previously evaluated in NSCLC clinical trials [26], ranked first among ten pairs involving EGFR(S752I759del). Its predicted *pK_d_* of 9.69 closely matched the experimental value of 9.72 (Figure 6C). Across all 96 pairs involving EGFR and its variants, canertinib, gefitinib, and erlotinib were the three highest-ranked distinct compounds.

The same analysis was applied to ABL1, the kinase component of the BCR–ABL1 fusion protein that drives chronic myeloid leukemia (CML) [27]. Among seven ABL1–ligand pairs, imatinib was ranked second, with predicted and experimental *pK_d_* values of 7.47 and 8.96, respectively (Figure 6D). The highest-ranked compound was PD173955 (CID 447077), an experimentally characterized Abl inhibitor [28], with corresponding values of 8.91 and 8.82. These compounds had the two highest experimental affinities in the subset, although their relative order was reversed in the predictions. The recovery of known inhibitors among the highest-ranked candidates supports the potential use of HierL2GDTA in prioritizing compounds for experimental screening against disease-associated targets.

## 3. Discussion

In this study, HierL2GDTA was developed to integrate local and global sequence information for drug–target binding affinity prediction. The framework first generates protein and molecular representations using pretrained language models. These representations are then processed through multi-scale local feature extraction, followed by distinct encoding mechanisms to capture broader contextual dependencies. Hierarchical attention-based fusion subsequently integrates global protein information and ligand features into the protein representation, connecting intramolecular dependency modeling with intermolecular interaction learning.

Benchmark evaluations on BindingDB and Davis showed lower prediction errors and stronger correlations with experimental affinities than the evaluated baseline methods. Ablation analyses supported the contributions of the feature extraction and fusion components, with protein-to-ligand cross-attention making a substantial contribution to predictive performance. Replacing both pretrained representations with one-hot encodings also reduced performance, supporting their joint use within the framework. Beyond benchmark evaluation, atom-level attention maps highlighted functional groups and aromatic substructures associated with protein binding, offering qualitative interpretations of the predictions. Retrospective screening further recovered known EGFR and ABL1 inhibitors among the highest-ranked candidates. Although the pretrained protein and molecular embeddings provide informative contextual representations, some physicochemical and binding-related information is only implicitly captured in the current framework. Incorporating complementary structural and interaction features in future work may enable a more comprehensive characterization of protein–ligand recognition. Interaction recovery metrics and adapted interface agreement measures will be investigated to assess the fidelity of modeled binding interfaces and explore their potential as prior-informed scoring signals to guide model training and optimization [23,24]. These extensions may facilitate the explicit modeling of key intermolecular interactions and further improve predictive performance. Together, these findings support hierarchical local-to-global representation learning for affinity prediction and indicate that HierL2GDTA can help prioritize compounds for screening against disease-associated targets.

## 4. Materials and Methods

### 4.1. Datasets

Two public drug–target affinity datasets, BindingDB [29] and Davis [30], were used to train and evaluate HierL2GDTA. Following the preprocessing protocol described previously [31], the original BindingDB dataset containing 3,048,285 records was processed as follows. First, records involving multiple protein chains were excluded, leaving 2,871,422 entries. Records with missing SMILES or protein sequences were then removed, resulting in 2,871,348 records. Next, *K_d_* values were restricted to the range of 0.01–10^7^ nM, with values reported using “<” or “>” approximated by dividing or multiplying the reported thresholds by 3.16, respectively, yielding 112,647 records. Filtering based on target organisms and assay temperatures retained 108,226 records. Specifically, records from mammalian and avian targets were retained, while viral targets were considered only if the assay temperature was between 33 and 40 °C. Furthermore, Protein sequences outside the range of 50–2560 residues were excluded, leaving 108,004 records. SMILES strings were subsequently canonicalized using RDKit (version 2026.09.1), and invalid SMILES or those exceeding 300 characters were removed, resulting in 106,738 records. Finally, duplicate protein–ligand records with identical SMILES and protein sequences were consolidated by taking the 20th percentile of their *K_d_* measurements. The resulting BindingDB dataset contained 69,856 unique protein–ligand pairs. The Davis dataset was obtained directly from a publicly available preprocessed benchmark, comprising 30,056 kinase–inhibitor pairs involving 442 kinase targets and 68 inhibitors. For both datasets, the binding affinity labels were derived from *K_d_* values. Specifically, *K_d_* values expressed in nM were converted to *pK_d_* using the following equation:

(1) $$pK_{d}=-\log_{10}\left(\frac{K_{d}}{10^{9}}\right)$$

The length distributions of the protein sequence and ligand SMILES strings were analyzed before model training. Protein sequences were limited to 1400 residues, covering approximately 95% of the proteins. Sequences exceeding this length were truncated. Given the relatively small variation in SMILES length compared with protein sequences, ligand SMILES strings were encoded without truncation. Table 6 summarizes the detailed dataset statistics. Data density was defined as the proportion of protein–ligand pairs with available affinity labels among all possible combinations. BindingDB exhibited a highly sparse affinity matrix with a density of 0.16%, whereas Davis had complete affinity label coverage (100%). Given these differences in data density, the model was trained separately on each dataset to evaluate its performance across different data distributions. Following TEFDTA [32], drug–target pairs were randomly divided into training, validation, and test sets at a ratio of 7:2:1 using a fixed random seed.

### 4.2. Model Construction

Given a protein–ligand pair, the protein sequence and ligand SMILES string are first encoded by the pretrained ESM-2 [33] and ChemBERTa [34] models, respectively. The resulting residue and token embeddings provide contextualized representations for subsequent feature extraction. Multi-scale local features are then extracted separately from the protein and ligand representations using three parallel 1D convolutional layers with kernel sizes of 1, 3, and 5. The outputs of the three convolutions are fused to integrate local patterns at different receptive fields. To further capture long-range dependencies, different feature extraction modules are designed for proteins and ligands. For ligand representation, three parallel dilated convolutions [35] with a kernel size of 3 and dilation rates of 1, 2, and 4 are applied. The different dilation rates enlarge the receptive fields and capture dependencies between distant SMILES tokens at multiple scales. For protein representation, a BiGRU [36] is employed to capture bidirectional dependencies among amino acid residues. Finally, a modified Transformer [37] encoder is designed for hierarchical intramolecular and intermolecular interaction learning. Specifically, sinusoidal positional encoding (SPE) is first added to the protein, pooled protein context, and ligand representations to preserve sequential information. The protein representation is then processed by multi-head self-attention to capture intramolecular dependencies among protein residues. The resulting features pass through two fusion blocks, each applying protein self-attention, protein-to-context cross-attention, and protein-to-ligand cross-attention in sequence. These operations progressively integrate residue-level features, pooled protein context, and ligand information. The final representation is aggregated by masked attention pooling and passed to a three-layer feed-forward neural network (FFN) for binding affinity prediction.

### 4.3. Pretrained Sequence Representation

Protein sequences and ligand SMILES strings are first encoded using the frozen pretrained ESM-2 and ChemBERTa models, respectively. The ESM-2 encoder contains 33 Transformer layers and 650 million parameters, producing 1280-dimensional contextualized residue embeddings. Given a protein sequence $P=\left(p_{1},p_{2},\ldots ,p_{L_{p}}\right)$, its representation is expressed as:

(2) $$E_{P}=\mathrm{ESM}2\left(P\right)=\left[e_{1}^{p},e_{2}^{p},\ldots e_{L_{p}}^{p}\right]$$

where *p_i_* denotes the amino acid residue at position *i*, and *L_p_* is the protein sequence length. $e_{i}^{p}\in R^{1280}$ represents the contextualized embedding of the *i*-th residue.

ChemBERTa is a Transformer-based molecular language model pretrained on large-scale SMILES data. Given a tokenized SMILES sequence $D=\left(d_{1},d_{2},\ldots ,d_{L_{d}}\right)$, ChemBERTa generates the token-level representation:

(3) $$E_{D}=\mathrm{ChemBERTa}\left(D\right)=\left[e_{1}^{d},e_{2}^{d},\ldots e_{L_{d}}^{d}\right]$$

where *L_d_* denotes the number of SMILES tokens and $e_{i}^{d}\in R^{384}$ represents the contextualized embedding of the *i*-th token.

A compact protein context is constructed by applying adaptive average pooling to the pretrained residue embeddings along the sequence dimension. This operation produces *L_c_* = 16 context vectors, denoted by $C\in R^{L_{c}\times 1280}$. After projection into the common feature space, these vectors serve as the keys and values in protein-to-context cross-attention.

### 4.4. Multi-Scale Local Feature Extraction

Separate convolutional modules extract local features from the protein and ligand embeddings. Each module consists of three parallel one-dimensional convolutions with kernel sizes of 1, 3, and 5. For an input representation *X* ∈ {*E_P_*,*E_D_*}, the feature map obtained using a convolutional kernel of size *k* is expressed as:

*H_k_* = *σ*(*Conv*1*D_k_*(*X*)), *k* ∈ {1, 3, 5} (4)

where *k* denotes the convolutional kernel size, *σ* denotes the Gaussian Error Linear Units (GELU) activation function. Each convolution produces 640 feature channels. The resulting three feature maps, *H*_1_,*H*_3_,*H*_5_, are subsequently concatenated along the channel dimension:

*H_multi_* = *Concat*(*H*_1_,*H*_3_,*H*_5_) (5)

Accordingly, the three parallel convolutions capture complementary local patterns with different receptive fields. The kernel of size 1 transforms features independently at each position, whereas kernels of sizes 3 and 5 aggregate information from neighboring positions. Concatenating the branch outputs combines local sequence patterns captured at different receptive-field sizes. The concatenated representation therefore contains 1920 feature channels and is further processed for subsequent feature extraction.

### 4.5. Long-Range Dependency Modeling

To capture broader contextual information, protein and ligand features are further processed using a BiGRU and parallel dilated convolutions, respectively. The ligand features $H_{\mathrm{multi}}^{D}$ obtained from the multi-scale convolutional module are processed by three parallel one-dimensional dilated convolutions, with a kernel size *K* of 3 and dilation rates *d* of 1, 2, and 4.

(6) $$H_{\mathrm{multi}}^{D}=\left[h_{1}^{D},h_{2}^{D},\ldots ,h_{L_{d}}^{D}\right]\in R^{L_{d}\times C_{\mathrm{in}}}$$

(7) $$h_{i}^{\left(d\right)}=\sigma \left(\sum_{m=0}^{K-1}W_{m}^{\left(d\right)}h_{i+d\left(m-\left\lfloor K/2\right\rfloor \right)}^{D}+b^{\left(d\right)}\right)$$

where $h_{i}^{\left(d\right)}$ denotes the output feature vector at position *i* for dilated rate *d*, *C_in_* is the input feature dimension. $W_{m}^{\left(d\right)}$ is the weight matrix, *b*^(*d*)^ is the bias term, and *σ* denotes the activation function. Appropriate padding is applied at sequence boundaries to preserve the sequence length. The receptive field of dilated convolution is calculated as:

*R* = 1 + (*K* − 1)*d* (8)

For a kernel size of 3, dilation rates of 1, 2, and 4 correspond to receptive-field spans of 3, 5, and 9 positions in the input feature sequence, respectively. The parallel branches therefore aggregate information over different sequence ranges without increasing the kernel size. Each of the three parallel dilated convolutions produces features with 640 channels. The outputs are concatenated along the channel dimension as follows:

(9) $$H^{\left(d\right)}={\mathrm{DilatedConv}}_{K=3,d}\left(H_{\mathrm{multi}}^{D}\right),d\in \left\{1,2,4\right\}$$

*H_dilat_* = *Contat*(*H*^(1)^,*H*^(2)^,*H*^(4)^) (10)

where $H^{\left(d\right)}=\left[h_{1}^{\left(d\right)},h_{2}^{\left(d\right)},\ldots ,h_{L_{d}}^{\left(d\right)}\right]$. The concatenated features *H_dilat_*, with a feature dimension of 1920, are subsequently projected to a common representation dimension of 384 for downstream interaction modeling.

For protein encoding, the BiGRU processes the residue features in both sequence directions to model long-range dependencies. The hidden states are updated through reset and update gates, as defined in Equations (11)–(14):

*z_i_* = *f*(*W_z_x_i_* + *U_z_h_i_*_−1_ + *b_z_*) (11)

*r_i_* = *f*(*W_r_x_i_* + *U_r_h_i_*_−1_ + *b_r_*) (12)

(13) $${\tilde{h}}_{i}=\tanh\left[W_{h}x_{i}+U_{h}\left(r_{i}⨀h_{i-1}\right)+b_{h}\right]$$

(14) $$h_{i}=\left(1-z_{i}\right)⨀h_{i-1}+z_{i}⨀{\tilde{h}}_{i}$$

here, *x_i_* denotes the input feature of the residue at position *i*, *h_i_*_−1_ is the hidden state from the previous position, and *h_i_* is the updated hidden state. *z_i_* and *r_i_* represent the update and reset gates, respectively, while ${\tilde{h}}_{i}$ denotes the candidate hidden state. *W_z_*, *W_r_*, *W_h_*, *U_z_*, *U_r_*, and *U_h_* are weight matrices. *b_z_*, *b_r_*, and *b_h_* denote the corresponding bias vectors. *f* and *tanh* denote the sigmoid and hyperbolic tangent activation functions, respectively, and ⨀ represents element-wise multiplication. In the bidirectional architecture, the protein sequence is processed independently in the forward and backward directions:

(15) $${\vec{h}}_{i}={\mathrm{GRU}}_{f}\left(x_{i},{\vec{h}}_{i-1}\right)$$

(16) $${\overset{\leftarrow }{h}}_{i}={\mathrm{GRU}}_{b}\left(x_{i},{\overset{\leftarrow }{h}}_{i+1}\right)$$

where ${\vec{h}}_{i}$ and ${\overset{\leftarrow }{h}}_{i}$ denote the 640-dimensional forward and backward hidden states, respectively. Finally, the two hidden states are concatenated to obtain a bidirectional contextual representation of dimension 1280 at position *i*.

### 4.6. Hierarchical Intramolecular and Intermolecular Interaction Learning

The protein, ligand, and pooled protein-context features are independently projected into a common feature space of dimension *d* = 384, followed by layer normalization and dropout. The resulting representations are denoted as $P\in R^{L_{p}\times d}$, $D\in R^{L_{d}\times d}$, and $C\in R^{L_{c}\times d}$, respectively. SPE is then added to each representation:

(17) $$\tilde{P}=P+{\mathrm{SPE}}_{p},\;\tilde{C}=C+{\mathrm{SPE}}_{C},\;\tilde{D}=D+{\mathrm{SPE}}_{d}\;$$

The protein representation is first processed by two stacked self-attention layers to model dependencies among residues. Queries, keys, and values are obtained through separate learnable projections, and multi-head scaled dot-product attention is applied with a protein padding mask.

(18) $$Q_{p}=\tilde{P}W_{Q}^{p},{\;K}_{p}=\tilde{P}W_{K}^{p},{\;V}_{p}=\tilde{P}W_{V}^{p}\;$$

(19) $$\mathrm{Attn}\left(Q_{p},K_{p},V_{p}\right)=\mathrm{softmax}\left(\frac{Q_{p}K_{p}^{T}}{\sqrt{d_{k}}}+M_{p}\right)V_{p}$$

*MHA*(*P*) = *Concat*(*head*_1_,…,*head_h_*)*W_O_* (20)

where $W_{Q}^{p}$, $W_{K}^{p}$, $W_{V}^{p}$, *W_O_* are the projection matrices. *d_k_* denotes the dimension of each attention head. *M_p_* is the protein padding mask. *h* denotes the number of attention heads.

The resulting protein features are processed by two stacked hierarchical fusion blocks, each comprising three attention layers applied sequentially: protein self-attention, protein-to-context cross-attention, and protein-to-ligand cross-attention. Following self-attention, the updated protein representation *P_s_* serves as the query, while the pooled protein context $\tilde{C}$ provides the keys and values:

(21) $$Q_{c}={P_{s}W}_{Q}^{c},{\;K}_{c}=\tilde{C}W_{K}^{c},{\;V}_{c}=\tilde{C}W_{V}^{c}\;$$

(22) $${\mathrm{Attn}}_{c}=\mathrm{softmax}\left(\frac{Q_{c}K_{c}^{T}}{\sqrt{d_{k}}}+M_{c}\right)V_{c}$$

where $W_{Q}^{c}$, $W_{K}^{c}$, $W_{V}^{c}$ are the projection matrices. *M_c_* denotes the padding mask for the pooled protein context. This asymmetric attention mechanism enables each residue-level protein feature to selectively retrieve complementary information from the compressed protein context.

The context-enhanced protein features *P_c_* subsequently serve as queries for protein-to-ligand cross-attention, with ligand features providing the keys and values. Each attention sublayer uses a residual connection followed by layer normalization [38,39]. This operation allows each protein position to selectively aggregate ligand information, thereby modeling intermolecular dependencies. The final hierarchical feature representation is aggregated using masked attention pooling [40], which computes a weighted sum over valid residue positions:

(23) $$e_{i}=W_{b}^{T}\tanh\left(W_{a}h_{i}^{H}+b_{a}\right)\;$$

(24) $$\alpha_{i}=\frac{\exp\left(e_{i}\right)}{\sum_{j:m_{j}=1}\exp\left(e_{j}\right)},\;m_{i}=1$$

(25) $$h_{\mathrm{init}}^{H}=\sum_{i:m_{i}=1}\alpha_{i}h_{i}^{H}$$

where $h_{i}^{H}$ denotes the *i*-th feature vector in the intermolecular hierarchical representation, *W_a_*, *W_b_*, and *b_a_* are learnable parameters, *m_i_* and *m_j_* are binary indicators of valid residues at positions *i* and *j*, respectively. All self-attention and cross-attention layers in the hierarchical interaction learning module have an output feature dimension of 384.

Finally, the intermolecular feature representation with a dimension of 384 is fed into a three-layer FFN to predict drug–target binding affinity.

## Figures and Tables

**Table 1:** Performance of HierL2GDTA on the training, validation, and test sets of BindingDB and Davis.

Dataset	Data Split	MSE ↓	MAE ↓	RMSE ↓	R^2^ ↑	Pearson ↑	*ρ* ↑
BindingDB	Training	0.308	0.351	0.555	0.909	0.956	0.933
	Validation	0.621	0.504	0.788	0.817	0.909	0.882
	Test	0.568	0.483	0.754	0.830	0.917	0.890
Davis	Training	0.146	0.210	0.383	0.817	0.912	0.728
	Validation	0.260	0.276	0.510	0.679	0.842	0.680
	Test	0.251	0.278	0.501	0.688	0.844	0.677

↑ indicates that higher values are better, while ↓ indicates that lower values are better.

**Table 2:** Performance comparison of HierL2GDTA with baseline methods on the BindingDB and Davis test sets.

Dataset	Methods	MSE ↓	MAE ↓	RMSE ↓	R^2^ ↑	Pearson ↑	*ρ* ↑
BindingDB	AttentionDTA	0.771	0.564	0.878	0.769	0.886	0.863
	DeepAffinity	0.796	0.615	0.892	0.761	0.875	0.855
	DeepDTA	0.826	0.636	0.909	0.752	0.877	0.850
	GraphDTA	0.722	0.565	0.850	0.784	0.893	0.877
	MRBDTA	0.765	0.601	0.875	0.771	0.885	0.864
	MutualDTA	0.759	0.571	0.871	0.772	0.880	0.866
	HierL2GDTA	**0.568**	**0.483**	**0.754**	**0.830**	**0.917**	**0.890**
Davis	AttentionDTA	0.284	0.291	0.533	0.647	0.820	0.663
	DeepAffinity	0.333	0.458	0.577	0.586	0.778	0.639
	DeepDTA	0.332	0.363	0.576	0.588	0.784	0.642
	GraphDTA	0.337	0.316	0.581	0.581	0.797	0.630
	MRBDTA	0.315	0.325	0.561	0.609	0.815	0.658
	MutualDTA	0.290	0.309	0.539	0.640	0.819	0.660
	HierL2GDTA	**0.251**	**0.278**	**0.501**	**0.688**	**0.844**	**0.677**

Within each metric column, the best value is shown in bold. ↑ indicates that higher values are better, while ↓ indicates that lower values are better.

**Table 3:** Performance comparison of HierL2GDTA with baseline methods across three independent random splits on BindingDB and Davis.

Dataset	Methods	MSE ↓	MAE ↓	RMSE ↓	R^2^ ↑	Pearson ↑	*ρ* ↑
BindingDB	AttentionDTA	0.661 (0.096)	0.509 (0.047)	0.812 (0.058)	0.803 (0.029)	0.900 (0.012)	0.880 (0.015)
	DeepAffinity	0.769 (0.029)	0.609 (0.023)	0.877 (0.016)	0.771 (0.010)	0.879 (0.004)	0.858 (0.008)
	DeepDTA	0.786 (0.042)	0.625 (0.019)	0.886 (0.024)	0.766 (0.014)	0.879 (0.004)	0.855 (0.006)
	GraphDTA	0.753 (0.032)	0.610 (0.040)	0.868 (0.018)	0.775 (0.008)	0.885 (0.008)	0.865 (0.010)
	MRBDTA	0.755 (0.039)	0.619 (0.042)	0.869 (0.022)	0.775 (0.011)	0.883 (0.006)	0.854 (0.015)
	MutualDTA	0.749 (0.021)	0.603 (0.034)	0.865 (0.012)	0.777 (0.006)	0.883 (0.006)	0.863 (0.008)
	HierL2GDTA	**0.590 (0.020)**	**0.497 (0.013)**	**0.768 (0.013)**	**0.824 (0.005)**	**0.912 (0.004)**	**0.888 (0.002)**
Davis	AttentionDTA	0.270 (0.019)	0.261 (0.027)	0.520 (0.019)	0.661 (0.022)	0.840 (0.018)	0.668 (0.004)
	DeepAffinity	0.290 (0.039)	0.361 (0.085)	0.537 (0.035)	0.636 (0.045)	0.803 (0.023)	0.652 (0.012)
	DeepDTA	0.302 (0.026)	0.338 (0.022)	0.549 (0.023)	0.621 (0.029)	0.799 (0.013)	0.650 (0.013)
	GraphDTA	0.300 (0.035)	0.325 (0.010)	0.547 (0.032)	0.624 (0.040)	0.804 (0.009)	0.640 (0.011)
	MRBDTA	0.297 (0.032)	0.318 (0.006)	0.544 (0.029)	0.627 (0.038)	0.805 (0.022)	0.652 (0.010)
	MutualDTA	0.286 (0.003)	0.300 (0.008)	0.535 (0.003)	0.640 (0.001)	0.813 (0.006)	0.656 (0.007)
	HierL2GDTA	**0.257 (0.015)**	0.279 (0.006)	**0.507 (0.014)**	**0.677 (0.019)**	0.834 (0.009)	**0.673 (0.004)**

Bold and underlined values indicate the best and second-best results, respectively. The standard deviations are given in parentheses. ↑ indicates that higher values are better, while ↓ indicates that lower values are better.

**Table 4:** Performance comparison of HierL2GDTA with baseline methods under a protein-based cold split on BindingDB and Davis.

Dataset	Methods	MSE ↓	MAE ↓	RMSE ↓	R^2^ ↑	Pearson ↑	*ρ* ↑
BindingDB	AttentionDTA	1.856	0.967	1.362	0.312	0.577	0.597
	DeepAffinity	2.162	1.101	1.470	0.199	0.482	0.505
	DeepDTA	2.005	1.007	1.416	0.257	0.550	0.557
	GraphDTA	2.302	1.124	1.517	0.147	0.440	0.465
	MRBDTA	2.019	1.049	1.421	0.252	0.531	0.544
	MutualDTA	2.382	1.215	1.543	0.117	0.388	0.397
	HierL2GDTA	**1.850**	**0.947**	**1.360**	**0.315**	**0.608**	**0.600**
Davis	AttentionDTA	0.359	0.329	0.600	0.421	0.652	0.587
	DeepAffinity	0.464	0.447	0.681	0.253	0.503	0.435
	DeepDTA	0.444	0.424	0.666	0.284	0.534	0.484
	GraphDTA	0.570	0.458	0.755	0.081	0.523	0.455
	MRBDTA	0.484	0.436	0.695	0.220	0.514	0.463
	MutualDTA	1.147	0.991	1.071	−0.849	0.411	0.356
	HierL2GDTA	**0.348**	0.332	**0.590**	**0.440**	**0.683**	**0.592**

Bold and underlined values indicate the best and second-best results, respectively. ↑ indicates that higher values are better, while ↓ indicates that lower values are better.

**Table 5:** Results of the parameter analysis.

Parameters	MSE ↓	MAE ↓	R^2^ ↑	Pearson ↑
One fusion blocks	0.271	0.289	0.664	0.839
Three fusion blocks	0.298	0.294	0.629	0.826
Four fusion blocks	0.253	0.269	0.686	0.838
Fusion block (1, 1, 2)	0.272	0.274	0.662	0.824
Fusion block (1, 2, 1)	0.269	0.267	0.665	0.831
*d*_model_ = 192	0.276	0.287	0.656	0.843
*d*_model_ = 288	0.257	0.266	0.680	0.838
*d*_model_ = 480	0.253	**0.264**	0.686	0.841
HierL2GDTA	**0.251**	0.278	**0.688**	**0.844**

The default HierL2GDTA configuration consists of two fusion blocks, a (1, 1, 1) fusion configuration, and *d*_model_ = 384. The best value in each metric column is shown in bold. ↑ indicates that higher values are better, while ↓ indicates that lower values are better.

**Table 6:** Summary of the datasets.

Datasets	Number of Proteins	Number of Ligands	Number of Interactions	Max Length for Proteins (Coverage)	Max Length for Ligands (Coverage)
BindingDB	1557	27,480	69,856	1400 (95%)	244 (100%)
Davis	442	68	30,056	1400 (95%)	81 (100%)

## Data Availability

All datasets used in this study were obtained from publicly available repositories. The BindingDB dataset (release 202604) was obtained from https://www.bindingdb.org/ (accessed on 20 October 2026), and the Davis dataset was obtained from the PaddleHelix distribution at https://baidu-nlp.bj.bcebos.com/PaddleHelix/datasets/dti_datasets/davis_v1.tgz (accessed on 20 October 2026). The resource codes are available at https://github.com/WangKLLab/HierL2GDTA (accessed on 20 October 2026).

## Supplementary Materials

The following supporting information can be downloaded at: https://www.mdpi.com/article/10.3390/molecules31213856/s1, Figure S1: Training and validation loss curves of HierL2GDTA across three independent random splits on BindingDB and Davis; Table S1: Model parameters; Table S2: Ligand atom importance rankings and binding interactions for four representative protein–ligand pairs; Table S3: Binding affinity prediction performance of HierL2GDTA across 12 protein families in the Davis test set; Table S4: Predicted and experimental binding affinities of compounds evaluated against selected EGFR variants and ABL1 in the Davis test set.
