# Supplementary material for: HierL2GDTA: Hierarchical Interaction Modeling with Local and Global Features for Drug–Target Affinity Prediction
Source: Molecules. 2026 Oct 26;31(21):3856. doi: 10.3390/molecules31213856 (PMC13765231; doi:10.3390/molecules31213856)
Supplement: Supplementary file 1 [file molecules-31-03856-s001.zip › molecules-4621534-supplementary.pdf]

HierL2GDTA: Hierarchical Interaction Modeling with Local and Global Features for Drug–Target Affinity Prediction

Kaili Wang [1,*], Yaoxiang Yin [1], Yanglan Gan [1], Wentao Dai [2] and Zhijun Fang [3,*]

[1] School of Information and Intelligent Science, Donghua University, Shanghai 201620, China
[2] NHC Key Laboratory of Reproduction Regulation, Shanghai Institute for Biomedical and Pharmaceutical Technologies (SIBPT), Fudan University, Shanghai 200237, China
[3] Institute of Trustworthy Embodied AI, Fudan University, Shanghai 200433, China
* Correspondence: kailiwang@dhu.edu.cn (K.W.); zjfang@fudan.edu.cn (Z.F.)

Table S1. Model parameters.

Parameters	Davis	BindingDB
Protein feature encoder	ESM-2 650M	ESM-2 650M
Ligand feature encoder	ChemBERTa-77M-MTR	ChemBERTa-77M-MTR
Maximum protein input length	1,400	1,400
Maximum ligand input length	81	244
Protein embedding dimension	1,280	1,280
Ligand embedding dimension	384	384
Convolutional channel dimension	640	640
Representation dimension (d_{model})	384	384
Prediction FNN dimensions	{384, 1,536, 768, 1}	{384, 1,536, 768, 1}
Number of BiGRU layers	2	2
Initial self-attention layers	2	2
Number of cross blocks	2	2
Number of attention heads	4	4
Protein kernel sizes	{1, 3, 5}	{1, 3, 5}
Ligand kernel sizes	{1, 3, 5}	{1, 3, 5}
Ligand dilation rates	{1, 2, 4}	{1, 2, 4}
Dropout	0.15	0.15
Optimizer	AdamW	AdamW
Activation function	GELU	GELU
Masked pooling method	Attention	Attention
Batch size	150	150
Epochs	70	100
Learning rate	0.0001	0.0001
Weight decay	0.0001	0.0001
Gradient clip norm	5.0	5.0
Early stopping patience	15	20
Data split ratio	7:2:1	7:2:1
Random seed	42	42

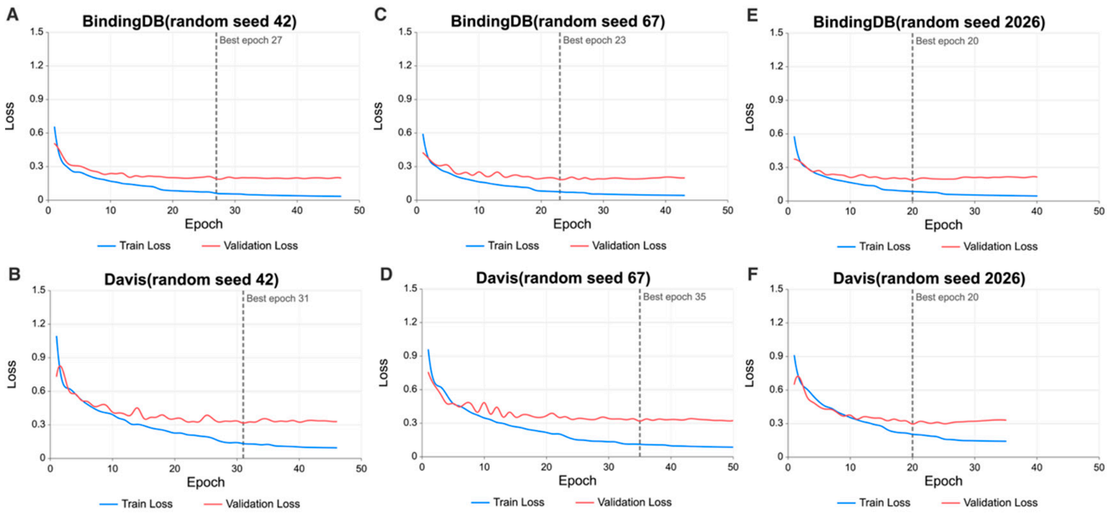

Figure S1. Training and validation loss curves of HierL2GDTA across three independent random splits on BindingDB and Davis.

Table S2. Ligand atom importance rankings and binding interactions for four representative protein–ligand pairs.

Protein–igand pair	Top 15 ligand atoms by importance	Interacting residues	Interactions
P00918–FSB	C20, C19, C4, N0, S1, F18, O3, O2, C17, C5, C16, C13, C14, C15, C12	H94(A), T199(A)	2 hydrogen bonds; 1 π–π stacking interaction
P42684–STI	N24, C25, N36, O7, C8, C23, C6, C9, N5, C10, C22, C11, C35, C12, N26	Y299(A), E332(A), T361(A), M364(A), I406(A), H407(A), D427(A)	5 hydrogen bonds; 2 π–π stacking interactions
ABL1–447077	C28, C12, C16, C15, C13, C17, C14, C25, C24, C26, C21, C9, N20, C8, O11	K271(A), M318(A)	2 hydrogen bonds; 1 cation–π interaction
KIT–5329102	C28, C26, O27, N25, F24, C23, C22, C18, C21, C19, C17, C20, N7, C8, C6	E671(A), C673(A)	2 hydrogen bonds

Table S3. Binding affinity prediction performance of HierL2GDTA across 12 protein families in the Davis test set.

Family	Targets	Pairs	MSE↓	MAE↓	R2↑	Pearson↑
class I PI3K	13	86	0.030	0.087	0.951	0.982
RET	4	31	0.206	0.303	0.886	0.944
FGFR	5	39	0.135	0.233	0.753	0.937
JAK	4	30	0.154	0.247	0.844	0.928
EGFR	15	114	0.275	0.274	0.839	0.916
PDGFR	18	131	0.388	0.371	0.813	0.914
CAMKL	19	116	0.167	0.219	0.795	0.908
RAF	3	21	0.469	0.424	0.694	0.904
CDK	17	113	0.159	0.237	0.750	0.885
Abl	16	108	0.325	0.342	0.725	0.865
STE20	27	188	0.202	0.256	0.721	0.857
Src	11	86	0.259	0.327	0.583	0.784

Table S4. Predicted and experimental binding affinities of compounds evaluated against selected EGFR variants and ABL1 in the Davis test set.

Targets	Inhibitors	Predicted affinity	Experimental affinity
EGFR(E746A750del)	CID 123631	8.647	9.268
	CID 208908	8.320	8.066
	CID 44259	6.825	6.921
	CID 10427712	5.675	5.387
	CID 153999	5.212	5.292
	CID 9929127	5.135	5.000
	CID 10138260	5.096	5.000
EGFR(L858R)	CID 176870	8.445	9.013
EGFR(L858RT790M)	CID 208908	8.320	5.000
EGFR(L858R)	CID 208908	8.320	8.553
EGFR(L858RT790M)	CID 3062316	6.619	5.658
EGFR(L858RT790M)	CID 11409972	6.219	5.796
EGFR(L858RT790M)	CID 11984591	6.213	7.215
EGFR(L858R)	CID 176155	6.057	5.721
EGFR(L858R)	CID 25243800	5.885	6.000
EGFR(L858RT790M)	CID 447077	5.710	5.000
EGFR(L858RT790M)	CID 153999	5.212	5.000
EGFR(L858R)	CID 5291	5.148	5.000
EGFR(L858RT790M)	CID 11717001	5.114	5.000
EGFR(L858RT790M)	CID 11617559	5.113	5.000
EGFR(L858RT790M)	CID 4908365	5.102	6.620
EGFR(L858RT790M)	CID 5329102	5.102	6.066
EGFR(L858R)	CID 10138260	5.096	5.000
EGFR(L858R)	CID 11712649	5.081	5.495
EGFR(L858R)	CID 5494449	5.053	5.000
EGFR(L858R)	CID 9926054	5.023	5.000
EGFR(L858R)	CID 24180719	5.011	5.000
EGFR(L858R)	CID 176167	4.981	5.000
EGFR(L858RT790M)	CID 11656518	4.980	5.000
EGFR(S752I759del)	CID 156414	9.688	9.721
	CID 208908	8.320	8.377
	CID 51004351	6.378	5.745
	CID 11984591	6.213	6.347
	CID 126565	6.209	5.854
	CID 5287969	5.938	5.854
	CID 25243800	5.885	5.337
	CID 16722836	5.535	5.000
	CID 11234052	5.284	5.000

	CID 9884685	4.966	5.000
ABL1	CID 447077	8.911	8.824
	CID 5291	7.466	8.959
	CID 123631	6.031	5.658
	CID 16007391	5.293	5.824
	CID 10461815	5.094	5.585
	CID 24889392	5.061	5.000
	CID 9926054	4.992	5.000
